# Dapagliflozin Alleviates Renal Fibrosis by Inhibiting RIP1-RIP3-MLKL-Mediated Necroinflammation in Unilateral Ureteral Obstruction

**DOI:** 10.3389/fphar.2021.798381

**Published:** 2022-01-07

**Authors:** Mei Ying Xuan, Shang Guo Piao, Jun Ding, Qi Yan Nan, Mei Hua Piao, Yu Ji Jiang, Hai Lan Zheng, Ji Zhe Jin, Can Li

**Affiliations:** ^1^ Department of Nephrology, Yanbian University Hospital, Yanji, China; ^2^ Department of Health Examination Central, Yanbian University Hospital, Yanji, China; ^3^ Department of Intensive Care Unit, Yanbian University Hospital, Yanji, China; ^4^ Department of Clinical Laboratory Medicine, Yanbian University Hospital, Yanji, China

**Keywords:** dapagliflozin, unilateral ureteral obstruction, necroinflammation, oxidative stress, endoplasmic reticulum stress, mitochondria

## Abstract

Dapagliflozin, a sodium-glucose cotransporter-2 inhibitor, offers renoprotection in diabetes. However, potential for use in nondiabetic kidney disease remains unknown. Herein, we assessed whether dapagliflozin alleviates renal fibrosis by interfering with necroinflammation in a rat model of unilateral ureteral obstruction (UUO) and *in vitro*. After induction of UUO, rats were administered dapagliflozin daily for seven consecutive days. UUO induced significant renal tubular necrosis and overexpression of RIP1-RIP3-MLKL axis proteins; these coincided with NLRP3 inflammasome activation, and subsequent development of renal fibrosis. Oxidative stress caused by UUO is tightly associated with endoplasmic reticulum stress and mitochondrial dysfunction, leading to apoptotic cell death through Wnt3*α/β*-catenin/GSK-3*β* signaling; all of which were abolished by both dapagliflozin and specific RIP inhibitors (necrostatin-1 and GSK872). In H_2_O_2_-treated HK-2 cells, dapagliflozin and RIP inhibitors suppressed overexpression of RIP1-RIP3-MLKL proteins and pyroptosis-related cytokines, decreased intracellular reactive oxygen species production and apoptotic cell death, whereas cell viability was improved. Moreover, activated Wnt3*α/β*-catenin/GSK-3*β* signaling was inhibited by dapagliflozin and Wnt/β-catenin inhibitor ICG-001. Our findings suggest that dapagliflozin ameliorates renal fibrosis by inhibiting RIP1-RIP3-MLKL-mediated necroinflammation via Wnt3*α/β*-catenin/GSK-3*β* signaling in UUO.

## Introduction

Globally, chronic kidney disease (CKD) causes increased socioeconomic burdens. Despite strict blood glucose control and use of hypotensive or antiproteinuric drugs, growth in the incidence of CKD and progress to end-stage renal disease continues unabated. According to national reports, the prevalence of CKD is proximately 14% in the United State and 10.8% in China (Zhang et al., 2012; Murphy et al., 2016). Renal fibrosis characterized by accumulation of extracellular matrix, infiltration of inflammatory cells, tubular epithelium cell apoptosis, and activation of fibroblasts, are the common signs of progressive CKD from virtually any etiology. Utilizing a well-known model of unilateral ureteral obstruction (UUO), we and others have defined important players, including oxidative stress, inflammatory mediators, transforming growth factor (TGF)-β1, and programmed cell death (Martínez-Klimova et al., 2019; Jin et al., 2020). Of these, oxidative stress-originated inflammation plays a critical role because it precedes ongoing renal scarring.

Necroptosis is a genetically regulated form of cell death modulated by receptor-interacting protein kinases one and 3 (RIP1 and RIP3, respectively) and downstream substrate pseudokinase mixed-lineage kinase domain–like (MLKL) (Choi et al., 2019). Dying necrotized cells release danger-associated molecular patterns (DAMPs), which subsequently activate innate immunity to evoke sterile inflammatory responses. The sterile inflammation in turn exacerbates necroptosis via two distinct pathways, via tumor necrosis factor-alpha (TNF-α) or interferon-gamma (Mulay et al., 2016). Driven by necrosis and inflammation, this autoamplification loop is referred to as necroinflammation. Emerging evidence demonstrates that RIP1-RIP3-MLKL-mediated necroinflammation is involved in the pathogenesis of obstructive nephropathy (Imamura et al., 2018; Popper et al., 2019), acute kidney injury (AKI) (Mulay et al., 2019), and the progression of AKI to CKD (Chen et al., 2018).

Sodium-glucose cotransporter 2 (SGLT2) inhibitors have been approved for the treatment of all stages of type 2 diabetes mellitus (T2DM). These drugs directly block SGLT2 and thereby inhibit renal glucose reabsorption, promote urinary glucose excretion, and effectively lower hyperglycemia. Because of these activities, SGLT2 inhibitors allow better blood glucose control compared with other antidiabetic agents beyond those with insulin-dependent actions. In addition, SGLT2 inhibitors have several advantages for patients with T2DM, including lower risk of hypoglycemia, reduced body weight (BW), and lower blood pressure (Verma and McMurray, 2018). Furthermore, clinical trials have demonstrated that SGLT2 inhibitors confer renoprotective and cardioprotective effects in patients with T2DM (Kubota et al., 2018; Neuen et al., 2019). Similar SGLT2 inhibitor renoprotective effects have been shown in animal models of renal ischemia/reperfusion injury (I/R) (Chang et al., 2016b; Rezq et al., 2020), gentamicin-induced nephrotoxicity (Mohamed et al., 2019), and chronic tacrolimus nephropathy (Jin et al., 2017). Nevertheless, the benefits of SGLT2 inhibitors in renal fibrosis of nondiabetic CKD have yet to be elucidated.

As such, we sought herein to assess whether dapagliflozin (Dapa) treatment affords renoprotection against renal fibrosis by inhibiting necroinflammation in a rat model of UUO and *in vitro*.

## Materials and Methods

### Experimental Groups and Treatment Protocol

Animal care and experimental procedures were reviewed and approved by the Animal Experimentation Ethics Committee of Yanbian University (SYXK [J]2020–0009) and the Animal Care Committee at the Medical College of Yanbian University (YBU-2019–11–27). Weight-matched male Sprague-Dawley rats weighing 240–260 g were housed in individual cages with a 12 h artificial light-dark cycle and permitted free access to standard chow and water. Following acclimatization for 1 week, rats were randomized into one of five groups and treated daily for 7 days: 1) sham group, sham operated rats without treatment; 2) UUO group, UUO rats without treatment; 3) UUO + Dapa group, UUO rats received Dapa treatment (10 mg/kg oral gavage, T2389/461,432–26–8, TargetMOI®) (Kapoor et al., 2015); 4) UUO + necrostatin-1 (Nec-1) group, UUO rats received Nec-1 treatment (2 mg/kg oral gavage, A4213, APExBIO) (Shen et al., 2019); 5) UUO + GSK872 group, UUO rats received GSK872 treatment (1 mg/kg intraperitoneal, HY-101872, MedChemExpress) (Guo et al., 2019). The UUO model was created as described previously (Jin et al., 2020). Briefly, rats were anesthetized with 1% pentobarbital sodium (40 mg/kg; Sigma-Aldrich) and a flank incision was made. After exposure of the kidney and ureter, the left ureter was ligated with 4–0 silk, and then the incision was sutured. Sham operations were like UUO, without ligation of the left ureter. Administration of Dapa, Nec-1, and GSK872 were started 24 h after UUO and continued for seven consecutive days. Rats were euthanized at the end of the study. Blood, urine, and kidney samples were rapidly collected for further examination.

### Biochemical and Functional Measurements

Body weight was monitored daily. At the end of the study, animals were placed individually in metabolic cages (ZH-B6, Anhui, China) and their water intake and urine volume were measured over a 24 h period. Fasting glucose levels (overnight) were measured from a drop of blood obtained from the tail vein, using a rapid glucose meter (ONETOUCH UltraVue, Johnson co, China). Urine protein excretion (UPE) was examined using enzymatic colorimetric methods (Roche Cobas 8,000 Core ISE, Roche Diagnostics, Hoffmann-La Roche Ltd, Basel, Switzerland). Renal function, serum lipid profiles, whole blood hemoglobin A1c (HbA1c), and high-sensitivity C-reactive protein (hs-CRP) were analyzed by an autoanalyzer according to the manufacturer’s instructions (Roche Cobas 8,000 Core ISE, Roche Diagnostics, Hoffmann-La Roche Ltd, Basel, Switzerland).

### Antibodies

The following antibodies were used: RIP1 (#53286, Cell Signaling Technology; 1:500), RIP3 (#ab62344, Abcam; 1:500), MLKL (#ab243142, Abcam; 1:500), interleukin-1beta (IL-1β, #ab9722, Abcam; 1:500), IL-18 (#ab191860, Abcam; 1:500), NOD-like receptor pyrin domain-containing protein 3 (NLRP3, #ab214185, Abcam; 1:200), ectodermal dysplasia-1 (CD68/ED-1, #ab125212, Abcam; 1:200), transforming growth factor-beta1 (TGF-β1, #ab179695, Abcam; 1:1000), connective-tissue growth factor (CTGF, #ab6992, Abcam; 1:500), 8-hydroxy-2′-deoxyguanosine (8-OHdG, JaICA, Shizuoka, Japan; 1:200), superoxide dismutase-2 (SOD2/MnSOD, #ab13534, Abcam; 1:1000), nicotinamide adenine dinucleotide phosphate oxidase 4 (NOX-4, NB110-58849, Product Datasheet, Novus Biologicals; 1:500), PINK1 (N4/15, #ab186303, Abcam; 1:500), Parkin (#2132, Cell Signaling Technology; 1:1000), p62 (#ab56416, Abcam; 1:500), succinate dehydrogenase complex subunit A (SDHA, #ab66484, Abcam; 1:1000), C/EBP homologous protein (CHOP, L63F7, #2895, Cell Signaling; 1:500), inositol-requiring protein-1α (IRE-1α, phospho S724, #ab37073, Abcam; 1:500), B-cell lymphoma-2 (Bcl-2, #ab196495, Abcam; 1:1000), Bcl2-associated X (Bax, #ab32503, Abcam; 1:1000), cleaved caspase-3 (#ab2302, Abcam; 1:500), Wingless-type MMTV integration site family member 3a (Wnt3a, #ab219412, Abcam; 1:500), glycogen synthase kinase-3beta (GSK-3β, #12456, Cell Signaling Technology; 1:1000), beta-catenin (β-catenin, #ab32572, Abcam; 1:1000), beta actin (β-actin, #ab8226, Abcam; 1:2000).

### Histopathological Examination

The kidney tissues were fixed in periodate-lysine-paraformaldehyde solution and embedded in wax. Following dewaxing, 4 μm sections were conducted and stained with hematoxylin-eosin (HE) and Masson’s trichrome. The quantitative analysis of fibrosis was performed using a color image auto-analyzer (VHX-7000, Leica Microsystems, Germany). A minimum of 20 fields per section was evaluated by counting the percentage of injured areas under ×100 magnification. Histopathological analysis was conducted in randomly selected fields of sections by a pathologist blinded to the assignment of the treatment groups.

### Immunohistochemistry

Immunohistochemical staining was performed as described previously (Jin et al., 2020). 8-OHdG and ED-1 were detected in 4 μm tissue sections with specific antibodies. Twenty different fields in each section at ×400 magnification were analyzed using a color image analyzer (VHX-7000, Leica Microsystems, Germany).

### Transmission Electron Microscopy

Transmission electron microscopy was performed as we previously detailed (Zhao et al., 2021b). Kidney tissues were post-fixed with 1% OSO4 and embedded in Epon 812 following fixation in 2.5% glutaraldehyde in 0.1M phosphate buffer. Ultrathin sections were cut and stained with uranyl acetate/lead citrate, and photographed with a JEM-1400Flash transmission electron microscope (JEM-1400Flash HC, JEOL Ltd, Tokyo, Japan). Using an autoimage analyzer, the number and size of mitochondria were measured in 20 random unoverlapped proximal tubular cells (VHX-7000, Leica Microsystems, Germany).

### Immunoblotting Analysis

Immunoblotting was fulfilled as described previously (Zhao et al., 2021b). Images were analyzed with an image analyzer (Odyssey® CL Imaging System, LI-COR Biosciences, NE, United States ). Optical densities were obtained using the sham group as 100% reference and normalized with *β*-actin.

### *In situ* TdT-Mediated dUTP-Biotin Nick End Labeling (TUNEL) Assay

Apoptotic cell death was identified using the ApopTag *in situ* Apoptosis Detection Kit (Sigma-Aldrich,Millipore). The number of terminal deoxynucleotidyl transferase-mediated dUTP nick-end labeling (TUNEL)-positive cells was counted on 20 different fields in each section at ×400 magnification.

### Enzyme-Linked Immunosorbent Assay (ELISA)

The urine concentration of the DNA adduct 8-hydroxy-2′-deoxyguanosine (8-OHdG) were measured using a competitive enzyme-linked immunesorbent assay (Japan Institute for the Control of Aging, Shizuoka, Japan) according to the manufacturer’s instruction. All samples were performed in triplicate and averaged.

### Cell Culture and Treatment

Human kidney proximal tubular epithelial cells (HK-2 cells) were obtained from the American Type Culture Collection (ATCC, Manassas, VA, United States ). HK-2 cells were grown in Dulbecco’s modified Eagle’s medium/Nutrient F12 (DMEM/F12; HyClone; GE Healthcare Life Science, Logan, UT, United States ) supplemented with 10% fetal bovine serum (FBS; Gibco; Thermo Fisher Scientific, Inc, Waltham, MA, United States ), 100 U/mL penicillin, and 100 μg/ml streptomycin (Gibco; Thermo Fisher Scientific, Inc, Waltham, MA, United States ). The cells were cultured in a humidified incubator with 5% CO2 and 37°C. Following 24 h incubation, cells were pretreated with or without different concentrations of Dapa (5 and 10 μmol/L) for 1 h and then coincubated with or without H_2_O_2_ (500 μmol/L), Nec-1 (30 mmol/L), GSK872 (3 μmol/L), and ICG-001 (10 μmol/L) for 24 h.

### Cell Viability Assay

The viability of HK-2 cells was evaluated using Cell Counting Kit-8 (CCK-8; Dojindo, Kumamoto, Japan) according to the manufacturer’s protocol. Approximately 1.0 × 10^4^ HK-2 cells/well were seeded in a 96-well plate. All groups of cells were treated as above described, then, 10 μL of CCK-8 solution was added to each well and incubated at 37°C for 3h. The absorbance was measured by determining the optical density at 450 nm (VersaMax Microplate Reader, Molecular Devices, LLC, Sunnyvale, CA, United States ).

### Measurement of Reactive Oxygen Species (ROS) Production

The levels of intracellular ROS production were measured using 2′, 7′-dichlorodihydrofluorescein diacetate (H2DCFDA, Invitrogen) according to the manufacturer’s instructions. HK-2 cells were seeded at a density of 2.0 × 10^5^ cells/well in a 6-well plate. All groups of cells were treated as above described, and then the cells were washed three times in PBS and incubated with H2-DCFDA for 30 min. The cells were washed and collected in PBS, and fluorescence was measured using a FACSCalibur flow cytometer (BD Biosciences, San Jose, CA, United States ).

### Apoptosis Assay

Annexin V-positive HK-2 cells were detected using an Annexin V-FITC apoptosis detection kit (Biosharp, Hefei, China) according to the manufacturer’s protocol. All groups of cells were treated as above described, and the cells were harvested, washed three times with PBS, and incubated with 1x binding buffer at a concerntration of 1 × 10^6^ cells/mL. Then, the cells were incubated with 5 μL of Annexin V-FITC and 5 μL of propidium iodide (PI) at room temperature for 15 min in the dark. The samples were analyzed within 1 h using a FACSCalibur flow cytometer (BD Biosciences, San Jose, CA, United States ). Apoptotic cells were determind as a percentage of the total cell count. The percentage of apoptotic cells was calculated as the number of PI-positive and Annexin-V-positive cells divided by the total number of cells. Three independent experiments were performed.

### Statistical Analysis

Data are expressed as mean ± SEM. Multiple comparisons between groups were performed using one-way ANOVA and the Bonferroni post hoc test using SPSS software (version 21.0; IBM, Armonk, NY). Statistical significance was assumed at *p* < 0.05.

## Results

### Effects of Dapa on Functional Parameters

BW loss was seen in all UUO7 groups, with or without drug treatment. Dapa treatment led to polyuria and increased WI during the experiment period. There were no significant differences in levels of UPE, blood lipid profiles and hsCRP within the experimental groups. Neither UUO nor Dapa and RIP inhibitors influenced renal function, as shown by Scr, BUN, and CysC (Table 1).

**TABLE 1:** Basic parameters in the experimental groups.

Parameters	Sham	UUO	UUO + Dapa	UUO + Nec-1	UUO + GSK872
ΔBW (g)	57 ± 2.7	38 ± 6.5^*^	40 ± 5.2^*^	37 ± 7.8^*^	40 ± 3.1^*^
WI (ml)	23 ± 1.8	25 ± 3.2	38 ± 2.7^*^	27 ± 3.0	21 ± 9.4
UV (ml)	18 ± 2.5	21 ± 1.9	35 ± 1.4^*^	23 ± 3.4	25 ± 2.6
FG (mg/dl)	107.0 ± 8.2	103.2 ± 11.5	91.2 ± 3.2	100.0 ± 6.1	92.1 ± 5.2
HbA1c (%)	3.6 ± 0.01	3.5 ± 0.03	3.8 ± 0.12	3.6 ± 0.04	3.4 ± 0.02
TG (mg/dl)	82.7 ± 17.5	78.3 ± 24.9	82.4 ± 20.1	76.6 ± 16.1	89.3 ± 15.4
CHO (mg/dl)	66.0 ± 4.6	71.0 ± 7.5	62.9 ± 8.6	64.5 ± 8.0	64.3 ± 3.6
HDL-C (mg/dl)	11.2 ± 0.7	12.3 ± 0.3	10.6 ± 0.8	10.2 ± 1.0	9.8 ± 2.0
LDL-C (mg/dl)	14.2 ± 1.1	16.5 ± 0.9	14.4 ± 1.6	15.5 ± 1.6	13.9 ± 0.8
hsCRP (mg/dl)	0.20 ± 0.03	0.21 ± 0.02	0.20 ± 0.03	0.18 ± 0.06	0.21 ± 0.04
UPE (mg/L)	318.8 ± 35.1	289.1 ± 40.6	319.0 ± 49.6	300.7 ± 38.7	301.8 ± 38.0
Scr (mg/dl)	0.30 ± 0.01	0.35 ± 0.02	0.28 ± 0.03	0.32 ± 0.02	0.34 ± 0.04
BUN (mg/dl)	132.8 ± 5.2	143.6 ± 12.3	139.3 ± 16.4	128.7 ± 9.0	142.5 ± 14.5
Cys-c (mg/L)	3.0 ± 0.20	2.9 ± 0.08	3.1 ± 0.13	2.8 ± 0.13	3.2 ± 0.16

Values are presented as mean ± SEM. UUO, unilateral ureteral obstruction; Dapa, dapagliflozin; Nec-1: necrostatin-1; ΔBW: body weight gain; WI: water intake; UV: urine volume; FG: fasting glucose; TG: triglyceride; CHO: cholesterol; HDL-C: high density lipoprotein cholesterol; LDL-C: low density lipoprotein cholesterol; hsCRP: high sensitivity C-reactive protein; UPE, urine protein excretion; Scr, serum creatinine; BUN, blood urea nitrogen; CysC, cystatin C. ^*^ *p* < 0.05 vs sham.

### Dapa Alleviates UUO-Induced Necroptosis

**FIGURE 1:**
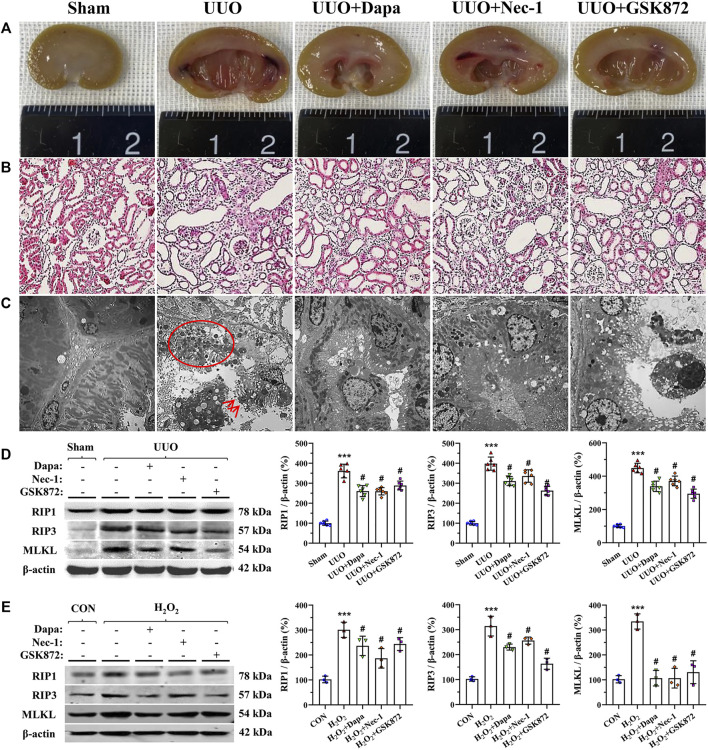
Representative photomicrographs of gross finding **(A)**, HE staining **(B)**, transmission electron microscopy **(C)**, and immunoblotting analysis of RIP1-RIP3-MLKL protein *in vivo* **(D)** and *in vitro* **(E)**. UUO induces a crowd of necrosis within the tubular epithelium (circle) and abscission of microvilli in epithelial cells (arrows), whereas these are improved following administration of Dapa or RIP inhibitor. Data are presented as mean ± SEM (*in vivo*, *n* = 6; *in vitro*, *n* = 3) and analyzed by one-way ANOVA. ^***^ *p* < 0.01 vs sham or control; ^#^ *p* < 0.05 vs UUO or H_2_O_2_.

Necroptosis has been strongly linked to the development of renal fibrosis in UUO (Xiao et al., 2017). Gross findings from digital images showed that UUO-induced pyelectasis with destruction of the pelvis and an extremely thin cortex, whereas these exterior changes were prevented by either Dapa or RIP inhibitors (Figure 1A). HE staining illustrated that UUO led to a significant renal tubular epithelial cell necrosis, tubular atrophy, and vacuolization (Figure 1B). Using electron microscopy, we clearly observed swelling of the tubular epithelium and interstitium, swarms of necrotized bodies, cytolysis, and abscission of microvilli in the tubular epithelial cell lumen of obstructed kidneys (Figure 1C). Dapa or RIP inhibitor (Nec-1 and GSK872) treatment alleviated these histopathological alterations compared with their untreated counterparts. Consistently, immunoblotting analysis showed that overexpression of RIP1-RIP3-MLKL axis proteins seen in the UUO group was significantly decreased by treatment of both Dapa and RIP inhibitors (Figure 1D).

To establish the rat model of UUO, we performed an *in vitro* study in HK-2 cells subjected to H_2_O_2_ treatment in the presence or absence of Dapa or RIP inhibitors. Consistent with the results of the *in vivo* study, either Dapa or RIP inhibitors suppressed RIP1-RIP3-MLKL axis proteins induced by H_2_O_2_ treatment (Figure 1E).

### Dapa Alleviates UUO-Induced Inflammation

**FIGURE 2:**
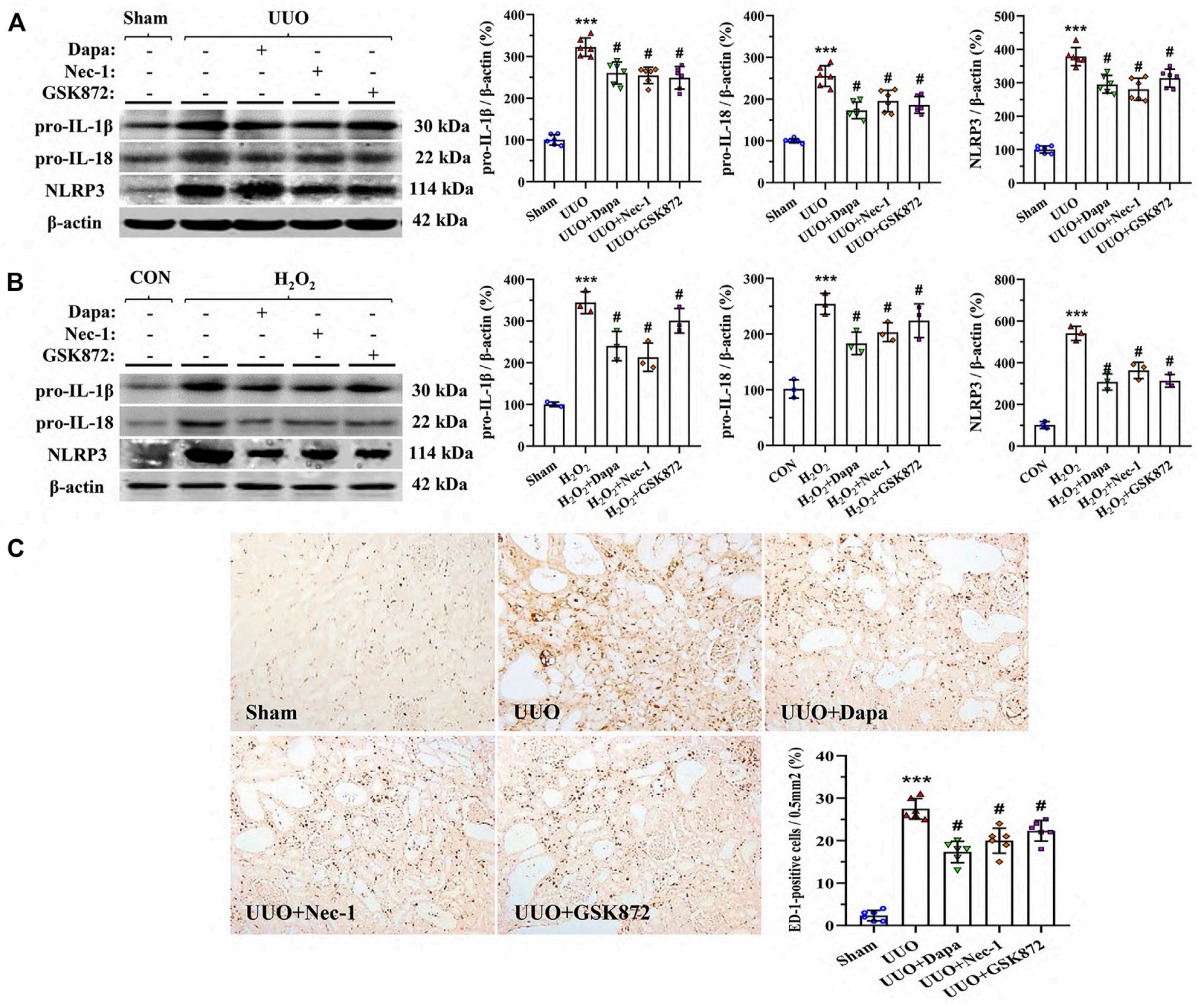
Representative photomicrographs of immunoblotting analysis of a series of pyroptosis-related proteins *in vivo* **(A)** and *in vitro* **(B)** and immunohistochemistry for ED-1 **(C)**. Data are presented as mean ± SEM (*in vivo*, *n* = 6; *in vitro*, *n* = 3) and analyzed by one-way ANOVA. ^***^ *p* < 0.01 vs sham or control; ^#^ *p* < 0.05 vs UUO or H_2_O_2_.

To define the effects of Dapa on interstitial inflammation caused by UUO, we studied the expression of pro-IL-1β, pro-IL-18, and NLRP3 based on previous studies (Gao et al., 2020; Zhao et al., 2021a). As shown in Figure 2A, UUO upregulated expression of pyroptosis-related cytokines (IL-1β, IL-18, and NLRP3), resulting in massive ED-1-positive cell infiltration (Figure 2C), whereas these were mitigated following Dapa or RIP inhibitors (Nec-1 and GSK872) treatment. In HK-2 cells, both Dapa and RIP inhibitors decreased expression of IL-1β, IL-18, and NLRP3 proteins (Figure 2B).

### Dapa Alleviates UUO-Induced Renal Fibrosis

**FIGURE 3:**
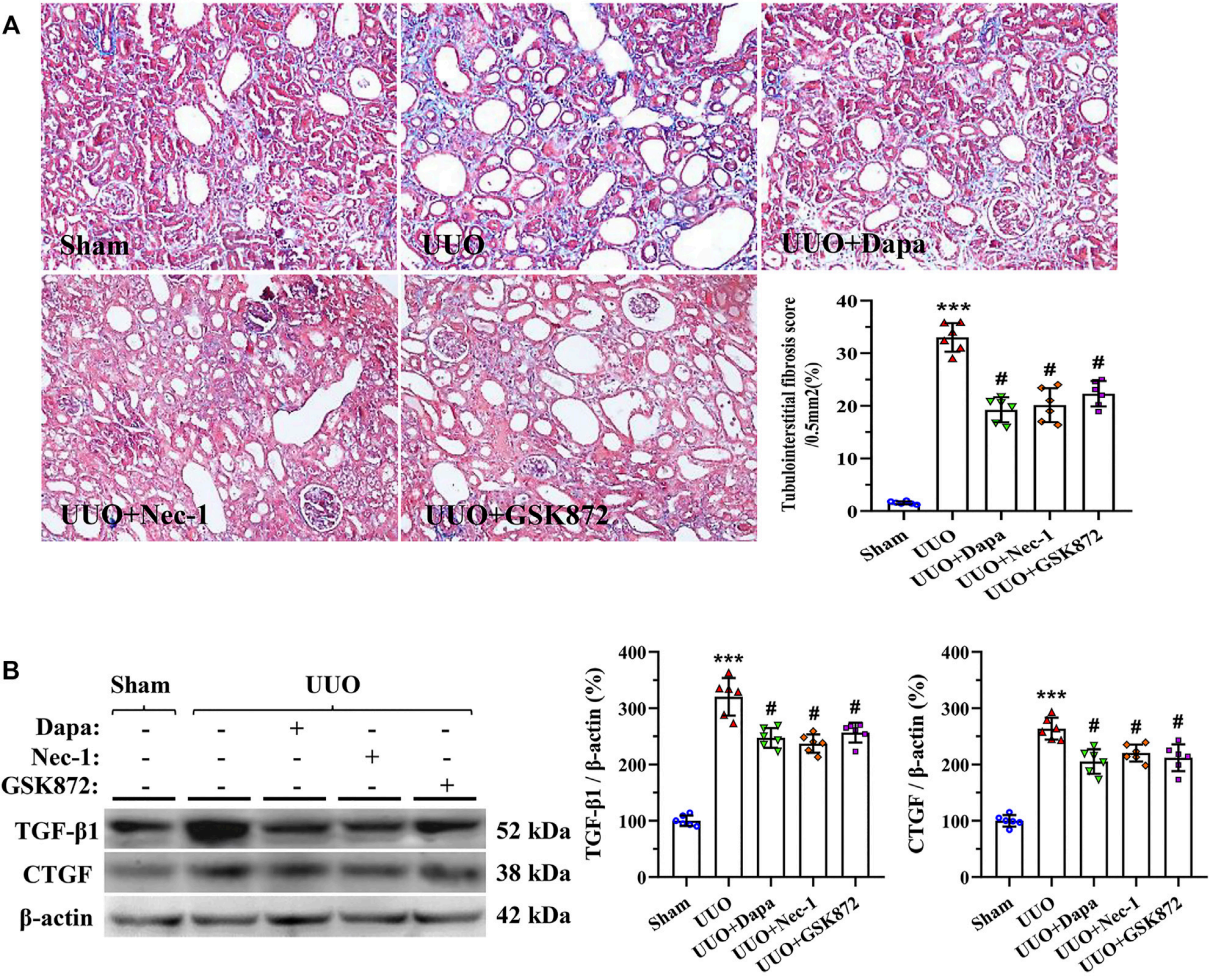
Representative photomicrographs of Masson trichrome staining **(A)** and immunoblotting analysis of TGF-β1 and CTGF **(B)**. Data are presented as mean ± SEM (*n* = 6) and analyzed by one-way ANOVA. ^***^ *p* < 0.01 vs sham; ^#^ *p* < 0.05 vs UUO.

Masson trichrome staining displayed that UUO-induced rat kidneys manifested by tubular vacuolization, collagen fibers deposition, and tubulointerstitial fibrosis (Figure 3A). Using our quantitative analysis system, increased fibrosis score in the UUO group was significantly decreased with treatment of Dapa or RIP inhibitors (Nec-1 and GSK872). Immunoblotting analysis revealed that either Dapa or RIP inhibitors dramatically inhibited profibrotic TGF-β1 and CTGF expressions compared with UUO alone (Figure 3B). Regarding the effect of Dapa and RIP inhibitors on necroptosis, sterile inflammation, and fibrosis, we suggest that Dapa alleviates renal fibrosis in this model of UUO may be associated with suppression of PIR1-PRI3-MLKL-mediated necroinflammation.

### Dapa Alleviates Oxidative Stress

**FIGURE 4:**
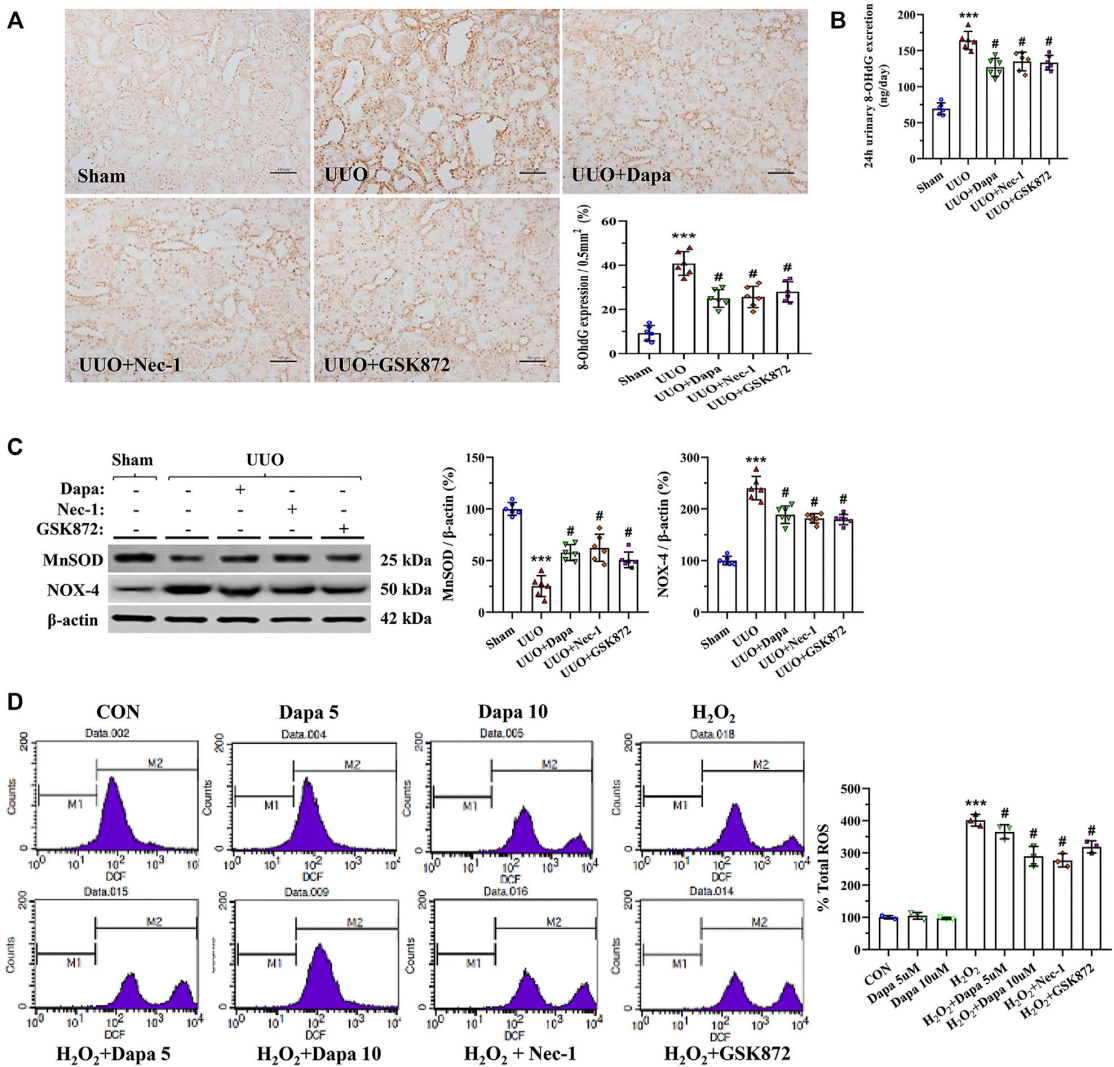
Representative photomicrographs of immunohistochemistry for 8-OHdG **(A)**, urine 8-OHdG concentration **(B)**, immunoblotting analysis of MnSOD and NOX-4 **(C)**, and intracellular ROS production in H_2_O_2_-treated HK-2 cells with or without Dapa and RIP inhibitors **(D)**. Data are presented as mean ± SEM (*in vivo*, *n* = 6; *in vitro*, *n* = 3) and analyzed by one-way ANOVA. ^***^ *p* < 0.01 vs sham or control; ^#^ *p* < 0.05 vs. UUO or H_2_O_2_.

Oxidative stress, necrosis, and inflammation are a vicious trio contributing to renal fibrosis during UUO, and an imbalance of oxidant and antioxidant enzymes may play a major role in this process. Figures 4A,B revealed that Dapa treatment significantly decreased 8-OHdG expression and urine 8-OHdG concentration in the UUO group. At a molecular level, Dapa counteracted oxidative stress by upregulating MnSOD expression but downregulated NOX-4 expression (Figure 4C). In HK-2 cells subjected to H_2_O_2_, Dapa dose-dependently decreased intracellular ROS production (Figure 4D).

### Dapa Alleviates UUO-Induced Mitochondrial Dysfunction and Endoplasmic Reticulum (ER) Stress

**FIGURE 5:**
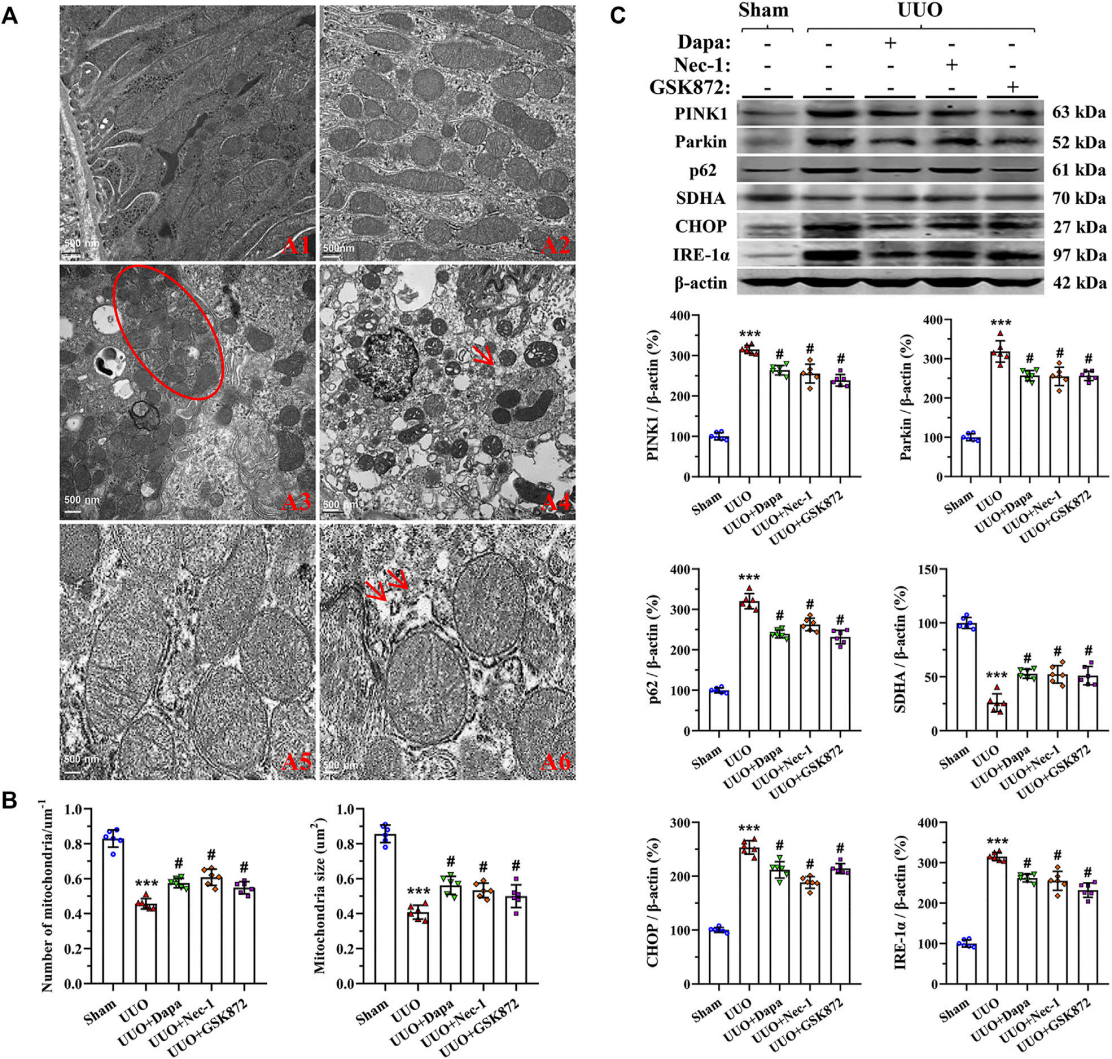
Representative photomicrographs of transmission electron microscopy of mitochondrial and ER morphology **(A)**, quantitative analysis of mitochondrial number and size **(B)**, and immunoblotting analysis of mitochondria- and ER stress-related proteins **(C)**. A1, normal mitochondria; A2, reduced mitochondria number and size and mitochondria fusion; A3, mitochondria divided into two or more organelles (circle); A4, autophagy of mitochondria (mitophagy, arrow); A5, normal rough ER; A6, degranulation of ribosomes and disconnected and dilated cisternae of rough ER (arrows). Data are presented as mean ± SEM (*n* = 6) and analyzed by one-way ANOVA. ^***^ *p* < 0.01 vs sham; ^#^ *p* < 0.05 vs UUO.

As delineated in Figure 5A, UUO damaged the mitochondrial architecture and fitness, characterized by a significant decrease in the number and size of mitochondria, dilatation of disorganized cristae, vacuolization, mitochondrial fusion, mitophagy formation, and mitochondria divided into two or more daughter organelles (fission). Quantitative analysis revealed that Dapa treatment preserved the number and size of mitochondria (Figure 5B). Immunoblotting showed that dysregulated expressions of PINK1, Parkin, p62, and SDHA in the UUO group were balanced by treating Dapa (Figure 5C). In addition, UUO was associated with degranulation of ribosomes, disconnected and dilated cisternae and peroxisome vacuolization in rough ER, whereas smooth ER remained almost normal in structure. Paralleled with the morphological findings, immunoblotting analysis revealed that UUO increased expression of ER stress-related genes such as CHOP and IRE-1α, but their expressions were decreased by Dapa administration (Figure 5C).

### Dapa Alleviates Apoptotic Cell Death

**FIGURE 6:**
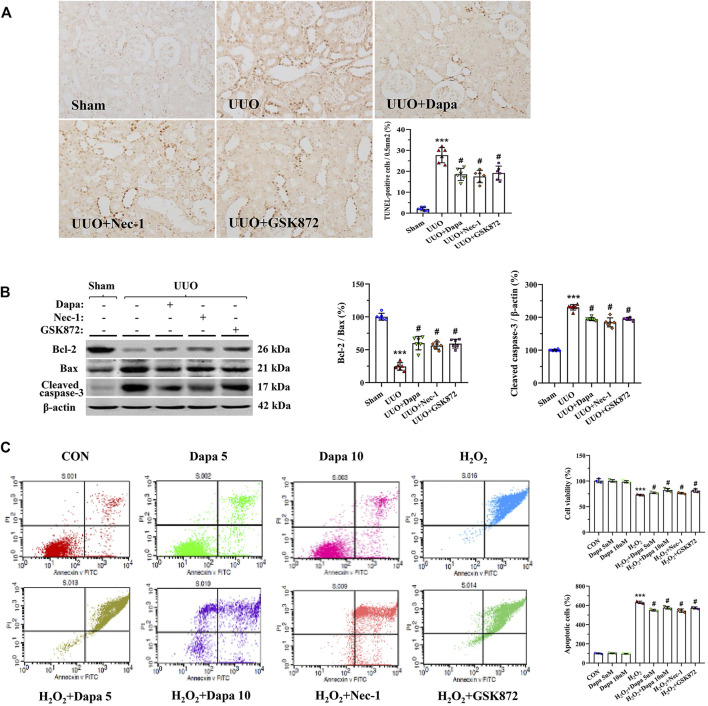
Representative photomicrographs of the TUNLE assay **(A)**, immunoblotting analysis of apoptosis-related genes **(B)**, and apoptotic cell death and cell viability in HK-2 cells **(C)**. Data are presented as mean ± SEM (*in vivo*, *n* = 6; *in vitro*, *n* = 3) and analyzed by one-way ANOVA. ^***^ *p* < 0.01 vs sham or control; ^#^ *p* < 0.05 vs UUO or H_2_O_2_.

Because of its pivotal role in UUO, we evaluated apoptotic cell death and expression of apoptosis-controlled genes in rat kidneys (Yang et al., 2018). Using the TUNEL assay, we found that most TUNEL-positive cells were in the tubular epithelial cells and interstitial cells, where tubular atrophy and fibrosis had developed (Figure 6A). Quantifying the number of TUNEL-positive cells revealed that an increase in their number in the UUO groups was significantly decreased in the UUO + Dapa group. Immunoblotting analysis showed that Dapa treatment regulated the Bcl-2/Bax ratio and cleaved caspase-3 expression trended toward cell survival (Figure 6B). In vitro study, Dapa dose-dependently improved cell viability and decreased apoptotic cells (Figure 6C).

### Dapa Inactivates Wnt3*α/β*-Catenin/GSK-3β Signaling

**FIGURE 7:**
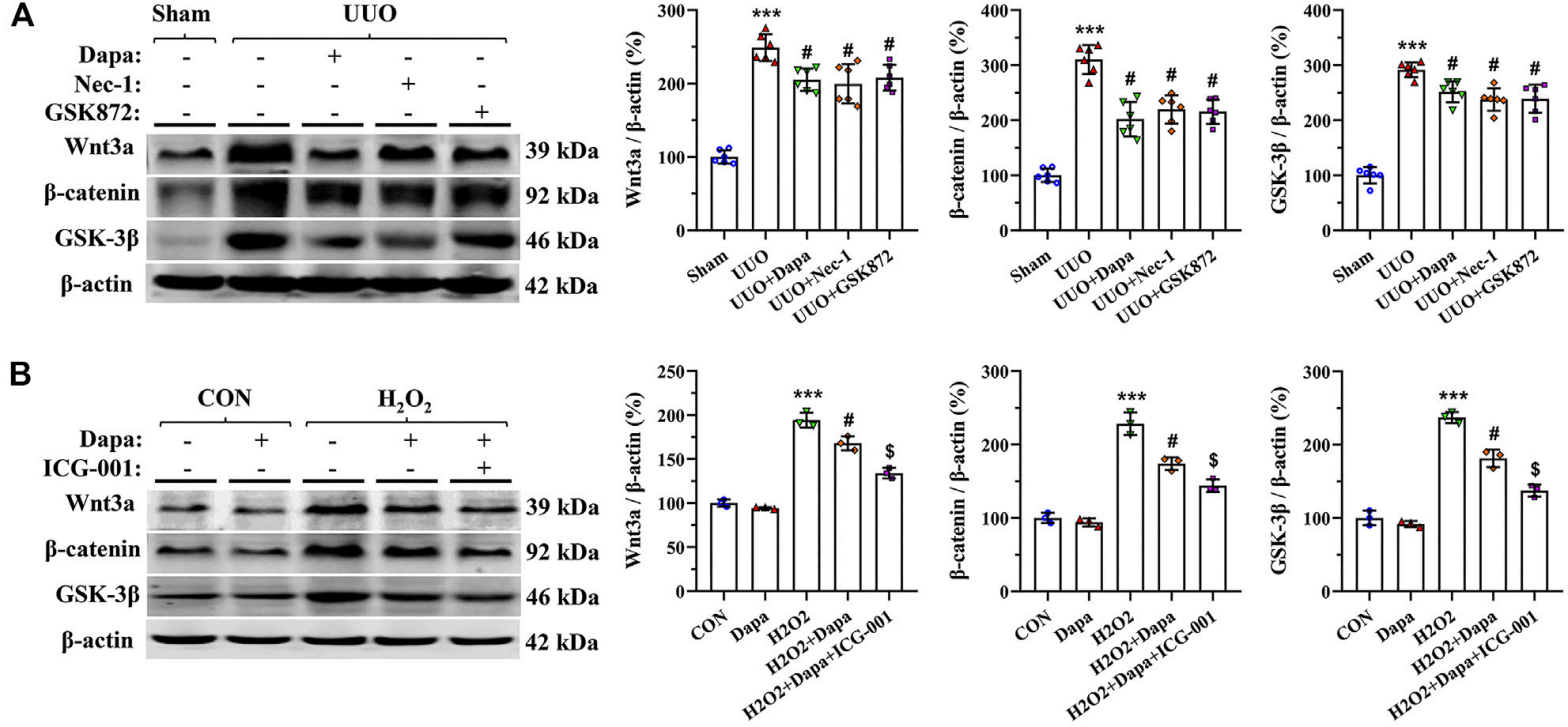
Representative photomicrographs of immunoblotting analysis of Wnt3*α/β*-catenin/GSK-3 *β* signaling proteins *in vivo* **(A)** and *in vitro* **(B)**. Data are presented as mean ± SEM (*in vivo*, *n* = 6; *in vitro*, *n* = 3) and analyzed by one-way ANOVA. ^***^ *p* < 0.01 vs sham or control; ^#^ *p* < 0.05 vs. UUO or H_2_O_2_; ^$^ *p* < 0.05 vs H_2_O_2_+Dapa.

We evaluated the effects of Dapa on expression of Wnt3*α/β*-catenin/GSK-3β signaling in a rat model of UUO and in H_2_O_2_-treated HK-2 cells by immunoblotting analysis. Figure 7 shows that both UUO and H2O2 activated Wnt3α/β-catenin/GSK-3β protein expression, whereas their expression was significantly inhibited by either Dapa or Wnt/*β*-catenin inhibitor ICG-001. This finding indicates that Dapa treatment alleviates renal fibrosis through the Wnt3*α/β*-catenin/GSK-3*β*-dependent signaling pathway.

## Discussion

Large clinical outcome trials have validated that SGLT2 inhibitors (Empagliflozin or Canagliflozin) improve renal outcomes, as defined by reducing the risk of deteriorated nephropathy, progression to macroalbuminuria, incidence of renal replacement therapies, and occurrence of doubling of serum creatinine in patients with T2DM (Wanner et al., 2016; Neal et al., 2017). Moreover, a double-blind, placebo-controlled trial enrolling patients from 116 research centers showed that Dapa decreases the urine albumin-to-creatinine ratio and slows kidney disease progression in patients with moderate-to-severe CKD (Pollock et al., 2019). The results herein also reveal that Dapa treatment improves damage to kidney architecture caused by UUO. However, there were no significant differences in FG or HbA1c levels among treatment groups. This implies that Dapa may confer renoprotective effects against nondiabetic CKD beyond hypoglycemia.

A unifying theory of necroinflammation consists of necroptosis and sterile inflammation, which are reciprocally enhanced in an autoamplification loop. Necroptosis regulated by RIP1, RIP3, and MLKL evokes innate immunity by releasing DAMPs via the ruptured plasma membrane. Of interest, RIP3-MLKL signaling facilitates inflammation by stimulating NLRP3 inflammasome and thereby regulates the processing and secretion of pro-IL-1β and pro-IL-18 into the mature and active cytokines via caspase-1-activating platforms (Anders and Muruve, 2011; Anders, 2016; Choi et al., 2019). Pharmacological blockade of necroptosis or RIP3 genetic deficiency attenuates UUO-induced inflammation and fibrosis (Xiao et al., 2017; Imamura et al., 2018). Herein, we found that UUO increases expression of RIP1-RIP3-MLKL proteins, which is accompanied by upregulation of pyroptosis-controlling genes and proinflammatory cytokines, and subsequent development of renal fibrosis. However, these effects were all reversed by either Dapa or RIP inhibitors. We propose that Dapa alleviates renal fibrosis by suppressing UUO-induced necroinflammation.

How Dapa treatment abrogates renal necroinflammation and fibrosis in this model is unclear but may be related to oxidative stress. It is well known that UUO is closely associated with hypoxia, which results in oxidative stress. Dapa directly inhibits cytosolic and mitochondrial ROS production in HK-2 cells subjected to H_2_O_2_ (Zaibi et al., 2021). However, Dapa also indirectly decreases ROS production and expressions of NOX-4 and NOX-2 through its hypoglycemic action in db/db mice and in mProx24 cells (Terami et al., 2014). A similar antioxidative effect of Dapa is also reflected in failing heat stroke (Olgar and Turan, 2019) and hepatic steatosis (Tang et al., 2017). Herein, we observed that Dapa decreased NOX-4 expression and reduced 8-OHdG concentrations, but increased MnSOD expression. These findings suggest that the abrogation of necroinflammation and fibrosis by Dapa seen herein may contribute to its antioxidant property.

Chronic hypoxia may aggravate necrotic effects, causing renal cellular injury by oxidative stress, mitochondrial dysfunction, and ER stress. Inversely, mitochondria are a major source of ROS, which is involved in regulation of cell apoptosis (Galvan et al., 2017). We have previously demonstrated that sustained or prolonged ER stress may be cytotoxic, leading to apoptotic cell death (Han et al., 2008). Thus, oxidative stress, mitochondrial dysfunction, and ER stress are interrelated as a vicious cycle in the pathogenesis of UUO-induced renal injury. Using electron microscopy, we clearly observed that Dapa shields mitochondrial and ER structural integrity in UUO-treated rat kidneys. These morphological changes were followed by regulation of an array of mitochondrial- and ER stress-controlling genes, resulting in weakened apoptosis. Our findings are consistent with studies by Chang et al. in renal I/R injury (Chang et al., 2016a) and Shibusawa et al. in diabetic nephropathy (Shibusawa et al., 2019) showing the capacity of Dapa on ER stress and apoptosis.

Our study demonstrates that treatment with Dapa (10 mg/kg/d) alleviates renal fibrosis from UUO by inhibiting necroinflammation, oxidative stress, and apoptosis. This is supported by a variety of nondiabetic CKD studies, such as adenine-induced CKD (Ali et al., 2019) and angiotensin II-dependent kidney damage (Reyes-Pardo et al., 2019). However, an interesting result was observed from subtotal (5/6) nephrectomy. Zhang et al. described that Dapa treatment at 1.0 mg/kg/d did not affect mortality, heavy proteinuria, or declining glomerular filtration rate (Zhang et al., 2016). Nor did Dapa attenuate the degree of glomerulosclerosis, tubulointerstitial fibrosis, or overexpression of TGF-ß1 mRNA in the kidneys of 5/6 nephrectomized rats. Moreover, Ma et al. reported that empagliflozin (10 mg/kg/d) has no effect on either renal dysfunction or profibrotic markers with oxalate-induced nephropathy on days 7 and 14 (Ma et al., 2017). The reasons for this discrepancy in the role of SGLT2 inhibitors in nondiabetic CKD are unknown, but may be related to the optimal dose used, disease model type, or treatment duration. Further studies are needed to resolve these questions.

It is well accepted that activation of Wnt/β-catenin signaling triggers chronic inflammation and overproduction of ROS (MacDonald et al., 2009). In addition, Wnt/β-catenin signaling works in a combination fashion with TGF-β signaling in the process of fibrosis, and TGF-β signaling can evoke expression of Wnt/*β*-catenin superfamily members, and vice versa (Guo et al., 2012). Thus, Wnt/*β*-catenin/GSK signaling is involved in the development of renal fibrosis in acute kidney injury (Pan et al., 2021) and diabetic nephropathy (Chang et al., 2018). In our model of UUO, the pathogenesis of renal fibrosis involves a complex network orchestrated by necroptosis, oxidative stress, inflammation, TGF-β1, and activation of Wnt/catenin signaling (Jiang et al., 2015; Dai et al., 2020; Jin et al., 2020). Inhibition of necroptosis or Wnt/catenin signaling alleviates UUO-induced renal fibrosis (Xiao et al., 2017; Gong et al., 2021), implying a relationship between necroptosis and Wnt/β-catenin signaling. Using *in vivo* and *in vitro* studies, we observed that overexpressed Wnt3α/β-catenin/GSK-3β protein in UUO rat kidneys and HK-2 cells was inhibited by either Dapa or ICG-001. Therefore, it is likely that Dapa affords renoprotective effects, probably by interfering with the Wnt3*α/β*-catenin/GSK-3*β* signaling pathway.

Clinical trials and animal studies have delineated that SGLT2 inhibitors possess pleiotropic effects beyond hypoglycemia. The present study demonstrates that Dapa alleviates renal fibrosis via decreased RIP1-RIP3-MLKL-mediated necroinflammation in a rat model of UUO. Suppression of oxidative stress, ER stress, and apoptosis, along with improved mitochondrial fitness, may be a mechanism underlying the renoprotective properties of Dapa. Our findings provide a potential rationale for the clinical use of SGLT2 inhibitors in preventing nondiabetic CKD.

## Data Availability

The original contributions presented in the study are included in the article/Supplementary Material, further inquiries can be directed to the corresponding authors.
